# Investigating the influence of visuospatial stimuli on driver’s speed perception: a laboratory study

**DOI:** 10.1186/s41235-023-00513-x

**Published:** 2023-09-13

**Authors:** Anna-Lena Köhler, Maren Klatt, Iring Koch, Stefan Ladwig

**Affiliations:** 1 Institute for Automotive Engineering (ika), RWTH Aachen University, Aachen, Germany; 2 Institute for Psychology, RWTH Aachen University, Aachen, Germany

**Keywords:** Speed perception, Safe speed, Traffic safety, Adaptive traffic intervention, Optic flow

## Abstract

Driving at an inappropriate speed is a major accident cause in the EU. Understanding the underlying sensory mechanisms can help to reduce speed and increase traffic safety. The present study investigated the effect of visuospatial stimuli on speed perception using an adaptive countermeasure to speeding based on a manipulation of optic flow. We added red lights on both sides of a simulated road. We expected speed to be perceived as faster when lights moved toward drivers due to increased optic flow, whereas we expected static light stimuli to not alter the optic flow and thus not influence speed perception. Two experiments applied the method of constant stimuli. To this end, participants encountered several trials of two video sequences on a straight road. A reference sequence showed the same traveling speed while test sequences varied around different traveling speeds. Participants indicated which sequence they perceived as faster, leading to the calculation of the point of subjective equality (PSE). A lower PSE indicates that the speed in this experimental condition is perceived as faster than in another experimental condition. Experiment 1A did not show a difference between PSEs of static and oncoming lights. Because participants had counted reflector posts for speed estimation, we removed these reflector posts in Experiment 1B and found a lower PSE for oncoming lights. Thus, such light stimuli may have an effect only in situations without other competing visual stimuli supporting speed perception. Future research should investigate whether speed perception is indeed a primarily visuospatial control task or whether other sensory information such as auditory factors can have an influence as well.

## Significance statement

Inappropriate speed remains a major cause of accidents. There is a variety of interventions aiming at lowering driving speed inappropriate for a given context. Countermeasures to speeding in the driving infrastructure can target every driver in potential areas. Understanding the mechanisms in speed perception can help to develop better interventions. The present two experiments investigated the role of visual stimuli in speed perception based on a dynamic light-based intervention that led to lower driving speeds in a previous simulator study and field test (Köhler et al., 2022). Our findings show that oncoming lights lead to lower perceived traveling speed, but only when there are no other salient visual stimuli that support speed perception. In sum, the findings provide valuable insights for future development of countermeasures targeting speeding.

## Introduction

Inappropriate speed is one major accident cause in the European Union, with 53% of fatalities having occurred on inter-urban roads in 2020 (European Commission, 2022) and traffic accidents still being the number one cause of death for children and young adults (World Health Organization, 2018). It is likely that accidents are multicausal events with a variety of potential causes and the human factor being one of them (Laaraj & Jawab, 2018). For instance, drivers likely often adopt traffic behavior that is inappropriate for the traffic situation, that is, inattention to possible risk or inappropriate speed (Karlsson et al., 2017). Furthermore, even a slight increase in speed could increase accident rates (Quddus, 2013) and, according to Elvik et al., (2004, 2019) and Hauer (2009), the higher the driving speed, the more inflexible could boundaries for action alternatives become. In this vein, this paper focuses on drivers’ speed perception as one influencing factor in traffic safety.

Causes for speeding are manifold and could either be conscious, ranging from person-related factors, such as attitude toward speeding, over situational factors, such as being late for an appointment, to social factors, such as peers challenging to drive faster (Wiafe et al., 2020). These aspects could be the reason that drivers might willingly decide to speed and thus enter a so-called dread zone, which means that they voluntarily leave their comfort zone where they feel safe (see Bärgman et al., 2015). According to the field of safe travel hypothesis (FoST; Gibson & Crooks, 1938; Papakostopoulos et al., 2017), drivers usually work toward staying safe and comfortable in traffic by keeping up safety margins between themselves and their surroundings. The FoST consists of the subjective impression where the driver feels save and the objective field, in which the vehicle can be operated safely (Gibson & Crooks, 1938). As there is no empirical evidence for the FoST according to the best knowledge of the authors, but it is mostly used to describe driver’s feeling of safety, Kolekar et al. (2020) operationalized the Driver’s Risk Field (DRF) in a study on obstacle avoidance. Results indicated that the DRF is wider than the vehicle itself. This emphasizes that the subjective perception of risk likely differs from objective parameters, which suggests that both areas should be investigated when it comes to traffic safety. According to Papakostopoulos et al. (2017), the FoST narrows along with the edges of the road, indicating that the driving environment and the cues that are available in it could potentially subliminally influence how drivers adjust their speed. This paper aims at investigating the role of cues in the driving environment on driver’s speed perception.

Horswill and McKenna (1999) conducted two video-simulation studies and found that visual and auditory information, even if distorted, were  the dominating modalities for processing information on speed, especially because drivers would tend to not look at their speedometers even if speed control would be required for the driving task. Driving speed decreased especially when internal car noise was raised. The relationship between objective information on speed and subjective speed perception has also found to be unstable in two speed estimation studies in a simulator and in a field test (Denton, 1980) and in a study on estimation of distances outside the driving context (Wu et al., 2004). This unstable connection between objective and subjective perception is a factor especially when it comes to the distortion of speed perception when slowing down after a long time traveling at higher speeds, which is called the speed adaptation effect (Denton, 1966). Stimuli in several perceptual modalities, such as proprioceptive (Kemeny & Panerai, 2003), vestibular (Macadam, 2003), or haptic information (Sigrist et al., 2013), can influence speed perception, with visuospatial stimuli likely having the biggest influence (see Köhler et al., 2022).

To facilitate safer traffic, the concept of self-explaining roads (SER) plays a role in designing a safe traffic environment (see Theeuwes, 2021). This means that road users know how to behave just by design of the driving environment. Selecting relevant information is crucial to successfully accomplish the driving task. Drivers have learned which objects to expect in the driving environment, making information selection efficient. In a nutshell, a well-designed road environment benefits the selection of appropriate and safe behavior. However, well-learned behavior can make drivers prone to bias and relevant cues can be overlooked, especially under high workload (Theeuwes, 2021).

In driving, a variety of information needs to be integrated by the driver’s information processing system at the same time to be able to accomplish the driving task, with speed control as one of several parallel tasks (Papakostopoulos et al., 2017; Stapel et al., 2019). Visual information has been described as one of the primary sources of information while driving (Macadam, 2003). According to Posner et al. (1976), visual attention needs to be allocated actively, requiring cognitive resources, and thus limiting processing input from other modalities. The latter is a potential reason for the advantage of visual stimuli in spatial tasks. As Gibson (1950) and Krüger et al. (1999) indicated, drivers are constantly monitoring both their own position and that of their vehicle in the driving environment. Therefore, speed control of a vehicle can be regarded a spatial control task (see also Köhler et al., 2022). This is supported by Ward et al. (2000), who stressed that vision is more prominent in spatial localization tasks than, for example, auditory information (see Lukas et al., 2010, 2014).

Another indicator for the dominance of visuospatial information in speed perception is the optic flow, which means that humans perceive their own driving speed relative to the speed of the environment (see, e.g., Gibson, 1950; Manser & Hancock, 2007; Palmisano, 2002). Among others, Ding et al. (2020) and Bergh Alvergren et al. (2019) used transverse line markings that move closer and closer together, thus creating the impression of becoming faster to decelerate drivers or cyclists, respectively. Both studies used visual cues in natural driving contexts, leading to lower driving speeds. Further, continuously decreasing spacing between transverse-bar road markings did lead to a reduction of mean speed by up to 4 mph (ca. 6.5 km/h) in a study by Gates et al. (2008). Therefore, the faster objects pass by, the faster speed is perceived. This has also been shown by Manser and Hancock (2007), who placed stripes on a tunnel wall that gradually decreased in width, and thus induced a feeling of acceleration. Palmisano (2002) found an effect of stereoscopic pattern on one’s own speed. Three-dimensional information about self-motion provided more information about driven speed than two-dimensional information and had the ability to radially expand optic flow, making illusions on optic flow more compelling. These countermeasures to speeding suggested that visuospatial stimuli have the potential to influence driver’s speed selection. To prevent situations in which drivers might not be aware of inappropriate speed, Köhler et al. (2022) conceptualized and studied a countermeasure to speeding by aiming at giving drivers an impression to be driving faster than they did, similar to the described studies with transverse line markings (Bergh Alvergren et al., 2019; Ding et al., 2020).

In a driving simulator study followed by a field test, Köhler et al. (2022) tested lights that were installed on both sides of the road, being activated if a driver approached at too high driving speeds. They investigated the influence of visual stimuli on speed adaptation in the form of red light-emitting spots. To prevent habituation effects (see, e.g., Hautzinger et al., 2011), the intervention was activated only for speeding drivers (see Köhler et al., 2022). Lights also have a higher stimulus salience than other static stimuli applied in the driving environment (see, e.g., Gates et al., 2008; Manser & Hancock, 2007; Palmisano, 2002). Choosing red lights was due to the color red being associated with stopping (Lidwell et al., 2010) and drivers reacting to red lights with caution in general (e.g., Edworthy & Adams, 1996; Norman, 1988). The sides of the road were chosen as a suitable location for a measure targeting driver’s speed perception when drivers might not be aware of driving too fast, because the sides of the road are usually in the driver’s periphery, which are exposed to faster motions (Zhang et al., 2013). The lights were then activated either moving toward the driver, thus highlighting the optic flow (e.g., Gibson, 1950), or were statically illuminated (non-moving). Participant’s speed adaptation when encountering either oncoming lights or static lights was compared to a baseline in a simulator study and in a subsequent field test by Köhler et al. (2022). A significantly lower speed for both static lights and oncoming lights, each compared to a baseline, was found in the field test, indicating that visuospatial cues in general can have an impact on driving speed, independently from the movement condition. The study’s results did, however, not reveal a significant difference in driving speeds between static lights and oncoming lights. It therefore remained open whether the presence of lights in general or one of the two movement conditions, static or oncoming lights, influenced driving speed more.

The present paper targets the influence of sensory factors on speed perception, specifically visual information, by investigating the sensory principles of the measure described by Köhler et al. (2022) more closely. The results of the field test by Köhler et al. (2022) suggested that participants drove more slowly when they encountered light stimuli, while the situation in the driving simulator was possibly not perceived to be critical enough to trigger a reaction by adjusting the driving speed as observed in the field test. Therefore, the present paper does not focus on actual speed selection as an indicator of speed perception but targets the influence of visuospatial stimuli on speed perception itself.

For this, we applied the method of constant stimuli (Hegelmaier, 1852, cited by Laming & Laming, 1992). In this method, various test stimuli or sequences are compared to a reference stimulus or sequence, which remains constant over the course of the study. The participant then has to decide and indicate via key press which stimulus they perceived as faster. Based on the dichotomous responses of the key press, a binary logistic regression is calculated for the independent variable and every participant. The results of this procedure can then be used to calculate the point of subjective equality (PSE). The PSE has been defined as the 0.5 probability point at which two stimuli look the same to an observer so that they would choose randomly between them (Kim et al., 2014). It is therefore a measure for the perceived driving speed, or traveling speed when participants are not driving themselves. Pretto et al. (2012), for example, used this method to examine the effects of fog on perceived traveling speed. We investigated the same light conditions as used by Köhler et al. (2022), namely red statically illuminated lights and red oncoming lights. We investigated the effect of the light stimuli on speed perception on a straight road instead of a motorway exit (as did Köhler et al., 2022), as this might influence speed selection since drivers usually slow down when approaching curves (Nash et al., 2016). We examined whether the addition of a movement component in the lights lead to a change in drivers’ speed perception. We expected the perceived speed in the condition with oncoming lights to be significantly higher than in the condition with static lights. This means that an objectively slower traveling speed in the condition with oncoming lights would be perceived to be as fast as an objectively higher traveling speed in the condition with static lights, in line with the optic flow (see Gibson, 1950; Manser & Hancock, 2007; Palmisano, 2002). Unlike Lidestam et al. (2019), who investigated virtual road markings, we tested light spots that were directly implemented on the road surface.

## Method

To test our hypotheses, we conducted two experiments, which we refer to as Experiment 1A and Experiment 1B. For practical reasons, we ran Experiment 1A before running Experiment 1B, but both experiments were completely comparable and we therefore report them as one between-subjects experiment.

### Sample

We used G*Power (Faul et al., 2007) to estimate our sample size a priori and assumed a medium effect of *d*_*z*_ = 0.5 (Cohen, 1988) based on the results of Pretto et al. (2012), who reported medium to large effects regarding the effect of visibility on visual speed. For our mixed design with the within-subjects factor *presentation of lights condition*, henceforth *presentation condition* (2; static lights vs. oncoming lights) and the between-subjects factor *presence of road markings* (2 levels; with road markings and reflector posts vs. without road markings and reflector posts), we calculated that a sample size of 28 participants for each experiment would allow us to detect an effect of this size at α = 0.05 with a power of 0.82 (see Faul et al., 2007). For a fully counterbalanced design, 28 participants took part in Experiment 1A of the study (*n* = 17 male, *n* = 11 female). The mean age was 25.11 years (*SD* = 4.52 years) with ages ranging from 18 to 44 years. 89.3% (*n* = 24) were right-handed. All participants had a normal or corrected-to-normal vision and held a driver’s license for a mean duration of 7.64 years (*SD* = 4.65 years), ranging from two to 26 years. The number of kilometers that was driven by the participants within a year ranged from 0 km to 30,000 km (*M* = 7694.64 km, *SD* = 7874.01 km). All participants were naïve to the purpose of the study and received a monetary compensation of 20 €.

For Experiment 1B, we recruited a larger sample, since it was part of a larger study, but with further considerations regarding power irrelevant for this paper. Thirty-six participants took part in Experiment 1B (*n* = 17 male, *n* = 19 female). This would have allowed us to detect a medium effect of *d*_*z*_ = 0.4 (Cohen, 1988). The mean age was 27.42 years (*SD* = 10.52 years) with ages ranging from 18 to 61 years. 88.9% (*n* = 32) were right-handed. All participants had a normal or corrected-to-normal vision, and 34 participants held a driver’s license for a mean 9.88 years (*SD* = 10.78 years), ranging from one to 44 years. The number of kilometers that was driven by the participants within a year ranged from 0 km to 44,000 km (*M* = 6377.06 km, *SD* = 8454.03 km). All participants were naïve to the purpose of the study. Participants in Experiment 1B participated voluntarily but did not receive any monetary compensation.

### Apparatus, task, and stimuli

We programmed the experiment on PsychoPy (2.0) and ran it on Windows10. Participants saw two video sequences per trial. Each trial consisted of a reference sequence and a test sequence. While the reference sequence remained the same over all trials (no light stimuli, traveling speed of 100 km/h), the test sequence varied in tested presentation condition (static or oncoming lights) and traveling speed in the video. We counterbalanced the order of test sequence and reference sequence among participants. For test sequences with static or oncoming lights, we placed lights every 6 m on the road surface to match the specifications of the simulator study by Köhler et al. (2022). As the lights, appearing as standard light studs used on normal roads, were placed directly on the road surface, they appeared smaller when being further away and grew in retinal size as they moved closer.

**Fig. 1:**
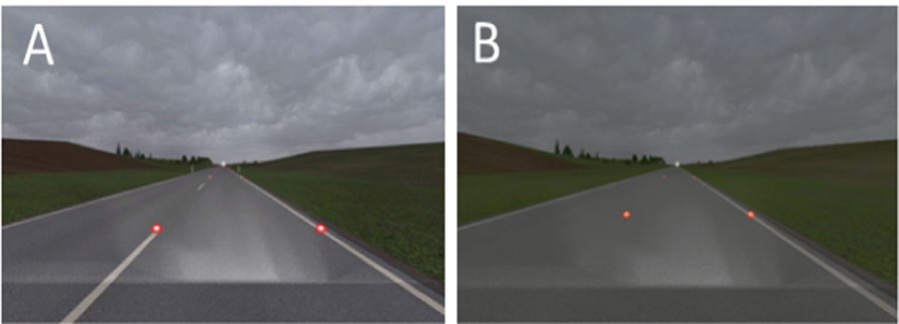
Exemplary screenshot of video sequences with red light stimuli and difference in road specifications between Experiment 1A and Experiment 1B of the study. *Note* Participants saw video sequences with red light stimuli on the roadside every 6 m that either remained static or were animated in a way to appear to be moving toward the driver. On the left **A** screenshot of the visual impression of Experiment 1A with reflector posts and road markings. On the right **B** screenshot of the visual impression of Experiment 1B without reflector posts and road markings

In Experiment 1A, the lane in the driving direction was separated from the opposing traffic lane by road markings (white broken line, as common on rural roads in Germany). Reflector posts stood on both sides of the road, see Fig. 1 part A. The distance between the posts was 50 m and therefore comparable to the distance of reflector posts on German roads. The starting point on the road was the same for all sequences. As we found that 21 out of 28 participants in Experiment 1A might have counted reflector posts to estimate speed, we decided to replicate the experiment (i.e., Experiment 1B), but remove reflector posts and road markings from the videos so that participants could focus solely on the light stimuli, see Fig. 1 part B. This allowed us to focus on the influence of the implemented visuospatial stimuli and assess the influence of the movement component of the visuospatial stimuli on speed perception. Not having road markings in the middle of the road is a common setup, for example, in rural roads in Germany, where the study was conducted.

Experiment 1A and Experiment 1B differed in the number of speed levels that participants experienced. However, this is not relevant for the determination of the PSE or differences in the PSE as a function of the independent variable and hence the PSEs are still fully comparable. In Experiment 1A, we used seven different traveling speeds in the test sequences, as five to seven levels are necessary to calculate the PSE properly (Becker-Carus & Wendt, 2017). Traveling speed in the test sequences with static lights varied from 70 to 130 km/h in 10 km/h steps to ensure an adequate range for calculating the PSE (Becker-Carus & Wendt, 2017). The range was scattered around 100 km/h, as traveling speed in the reference sequence was set to 100 km/h. We adjusted the speed levels in the condition with oncoming lights to vary between 50 and 110 km/h, again in 10 km/h steps. We followed this approach based on the simplified idea of the perceived speed to be a result of a linear relation of the actual personal (traveling) speed and the speed in which objects in the environment approach and roll by. We conducted a pretest with three participants for a first indication where the PSE might be located when encountering oncoming lights. The pretest suggested that the PSE would likely be around 80 km/h. Based on this information, we scattered the traveling speeds in the condition with oncoming lights around 80 km/h. This resulted in a range of 50 km/h to 110 km/h to avoid a ceiling effect of the test sequence always being indicated to be “faster” than the test sequence, which would have prevented the possibility to calculate a PSE. We tested each level of the factor presentation condition (the static lights and the oncoming lights) 20 times for each speed level to have enough answered trials to calculate the PSE. Thus, each participant encountered 280 trials, divided into 10 blocks à 28 trials to allow for breaks if needed.

In Experiment 1B, we used five different traveling speeds in the test sequences. We made this change to Experiment 1A, where we had tested seven different traveling speeds but found that five levels would be sufficient. An uneven number of speed levels was required to have the same number of speeds above and below the speed of the reference sequence. Thus, traveling speed in the test sequences with static lights ranged from 80 to 120 km/h in 10 km/h steps. As we found the line of argumentation to adjust the speed levels for oncoming lights to avoid a ceiling effect in responses in Experiment 1A to be valid, the speed levels for oncoming lights ranged from 60 to 100 km/h. Table 1 illustrates the tested speed levels in Experiment 1A and Experiment 1B. We tested each level of the factor presentation condition 30 times for each traveling speed, since fewer traveling speeds to be tested allowed for more repetitions of each traveling speed for a similar duration as in Experiment 1A, giving a higher probability to calculate a PSE for each participant. Thus, each participant encountered 150 trials, divided into 5 blocks à 30 trials. The trials were presented randomly across presentation conditions.

**Table 1:** Traveling speeds in video sequences for reference and test sequences

Experiment 1A			Experiment 1B		
Reference sequence without lights (km/h)	Test sequence with static lights (km/h)	Test sequence with oncoming lights (km/h)	Reference sequence without lights (km/h)	Test sequence with static lights (km/h)	Test sequence with oncoming lights (km/h)
100	70	50			
100	80	60	100	80	60
100	90	70	100	90	70
100	100	80	100	100	80
100	110	90	100	110	90
100	120	100	100	120	100
100	130	110			

We tested seven different speed levels in Experiment 1A and five different speed levels in Experiment 1B, since Experiment 1A showed that 5 levels would have been sufficient for calculating the PSE

**Fig. 2:**
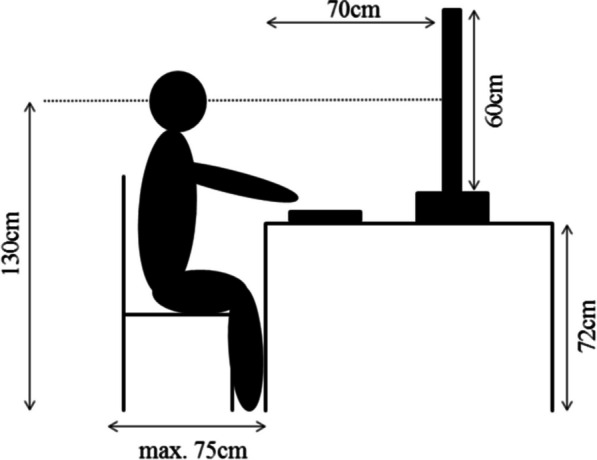
Setup and dimensions of experimental setup. *Note* Illustration of the participant sitting on a height-adjustable chair in front of a table holding the keypad and the screen. Heights and distances are illustrated

We compiled the video material for the tested sequences by recording footage from a driving simulator; the simulation was modeled by Virtual Test Drive 2.1 (by VIRES). VIRES road network editor is based on OpenDRIVE road networks. By using the test mode of Virtual Test Drive 2.1, we simulated a drive in the artificial environment with a constant speed according to the specified 10 km/h steps between 50 km/h and 130 km/h. We recorded the videos in MP4 format (HD 1080p 25 fps, 1920 × 1080). Bridges et al. (2020) tested a 60 Hz refresh rate for PsychoPy under Windows and achieved a mean precision in timing of 1 ms. We therefore assumed that PsychoPy can reliably render our 25 Hz videos and would have no problem with 60 Hz videos either. We recorded 3000 ms (ms) long video sequences that showed a section of a country road surrounded by a rural environment with a few trees in the distance (see Fig. 1). The duration of 3000 ms was longer than in other comparable studies, which used, for example, video sequences with a duration of 700 ms (Pretto et al., 2012) for the method of constant stimuli. Pilot testing prior to the experiment showed that the condition with oncoming lights required more time than 700 ms to create the impression of a movement. The duration of 3000 ms provided a subjectively good solution to see the movement properly. The condition with static lights was matched to this. The perspective in the video sequences was from the driver’s view and contained a fixation cross in the middle of the road on the level of the horizon. We standardized weather and light conditions to a cloudy sky just before dusk to simulate rather low-light conditions to have the red lights appear more prominent, see Fig. 1. The video sequences did not contain engine noises or auditory stimuli to prevent any sound-related effects. There was no oncoming traffic to prevent any influence on speed perception. We conducted both experiments in a closed test room at the Institute for Automotive Engineering of RWTH Aachen University, Germany. We blocked out daylight to ensure comparable light conditions. Participants were sitting on a height-adjustable chair in front of a desk on which a 49″ screen (Samsung 49″ Flat QLED 4k with 60 Hz frame rate) and a standard keyboard were placed in front of them. They adjusted the height of the seat for their eye level to be at the level of the center of the screen (Fig. 2) and sat centrally in front of the screen. The distance between the back of the chair and the desk did not exceed 75 cm. This ensured a standardized viewing angle and distance to the screen. We glued small patches of fleece fabric on the relevant keys Y (indicating the first video sequence to be perceived as faster) and M (indicating the second video sequence to be perceived as faster) so that participants could detect and select them easily.

We conducted a pre- and post-questionnaire to gather demographic data and additional information whether participants used strategies in the study and if so, which ones (multiple answers possible), and whether they had noticed the lights at all.

### Procedure

**Fig. 3:**
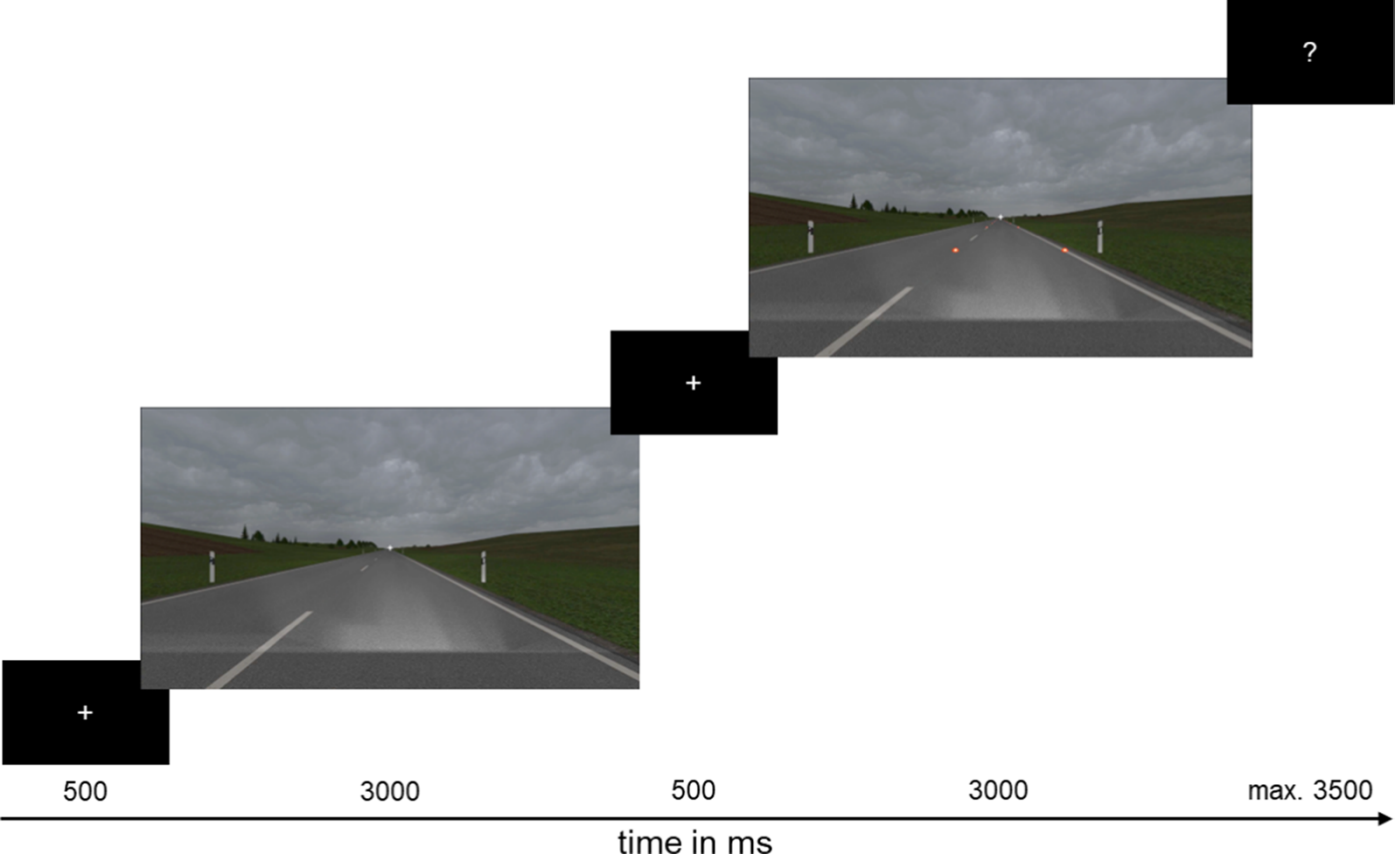
Trial Sequence. *Note* Illustration of the trial sequence with 500 ms of a black screen displaying a fixation cross, followed by a 3000 ms-long video sequence (reference sequence or test sequence, depending on the randomization), a 500 ms-long inter-stimulus interval, followed by another 3000 ms-long video sequence (reference sequence or test sequence, depending on the randomization), and a black screen with a question mark to indicate participants to respond, displayed for a maximum duration of 3500 ms. Video sequences in this figure show material from Experiment 1A

We conducted Experiment 1A in January 2020 and Experiment 1B in July 2021.^1^ Participants signed formal documents including giving informed consent to the study and completed a pre-questionnaire. We then asked them to adjust their sitting position and height according to the specifications and informed participants about the task of the study. Instruction of the task was presented on the screen. We informed participants that they were going to see two consecutively presented video sequences in each trial. When a question mark appeared on the screen, they had to rate if they perceived the traveling speed in the first or the second video as faster. We stressed that the decision relied solely on participants’ subjective impression, and there were no wrong responses. We set the maximum reaction time to 3500 ms to collect participant’s impulsive reactions. If participants did not respond, the next trial started automatically after expiry of the time limit. Figure 3 illustrates the trial sequence.

We informed the participants that the decision might be obvious in some trials but more difficult in other trials. In case of doubt, they were encouraged to make an intuitive decision. The reason for this is that the 0.5 probability level is essential for further analysis of the PSE: If people tended to rather press no key when being indecisive, it would have led to data loss and therefore problems when trying to determine the PSE. Breaks could be taken as needed between blocks. We told participants to focus on the fixation cross in the center of the screen. After the instruction, participants started with a test block of 10 trials. They were aware that the responses of the test block were not analyzed. To make sure participants fully understood the task, the instructor stayed inside the test room during the test block and answered remaining questions. After doing so, the instructor left the room and reentered when the study was completed. Experiment 1A took about 50 min, depending on how long participants’ breaks were. Experiment 1B was part of a larger study consisting of two further experimental blocks. Those included auditory stimuli corresponding to the light stimuli and are not part of this paper. This overall study followed a blocked approach to avoid condition order effects within one block. The order of blocks was randomized among the participants, including short breaks before another block started. Blocks were independent from one another. This paper focuses only on the block which replicated Experiment 1A, excluding reflector posts and road markings, to draw a direct comparison. The overall study lasted 75 to 80 min, with the block relevant for Experiment 1B taking about one-third of the overall time. The study ended with the post-questionnaire and a debriefing about the hypotheses of the experiment.

### Design

We based the study on a 2 × 2 mixed design with the within-subjects factor *presentation condition* (2 levels; static lights vs. oncoming lights) and the between-subjects factor *presence of road markings* (2 levels; with road markings and reflector posts vs. without road markings and reflector posts). The dependent variable was the PSE in kilometers per hour (km/h). Significance was tested at α = 0.05.

## Results

### Data preparation

To determine the PSE, binary logistic regressions were calculated for the data of both presentation conditions of every participant. We therefore calculated 56 binary logistic regressions for Experiment 1A and 72 binary logistic regressions for Experiment 1B due to the larger sample. We used the respective function with the coefficients α and β to calculate the PSE, which is the 0.5 probability point of the logistic function (Kim et al., 2014). For this, variables α and β of the respective logistic regression were inserted into the equation. As a prerequisite for calculating the PSE, each binary logistic regression must yield a significant result to assume a significant prediction between the predictor variable speed and the key response as outcome variable. For this reason, data sets from eight participants in Experiment 1B, with each having at least one non-significant result in one of the two calculated binary logistic regressions, were excluded, resulting in 28 participants for further analysis. We used IBM SPSS Statistics 24.0 for all calculations.

### Data analysis

We conducted a 2 × 2 mixed ANOVA with repeated measures on the within-subjects factor presentation condition (2 levels; static lights vs. oncoming lights) and the between-subjects factor presence of road markings (2 levels; with road markings and reflector posts vs. without road markings and reflector posts). The ANOVA revealed a significant main effect for presentation condition, *F*(1,54) = 8.50, *p* = 0.005, η_p_^2^ = 0.14, a significant main effect for presence of road markings, *F*(1,54) = 17.44, *p* < 0.001, η_p_^2 ^= 0.24, and a significant interaction between presentation condition and presence of road markings, *F*(1,54) = 8.94, *p* = 0.004, η_p_^2 ^= 0.14.

**Fig. 4:**
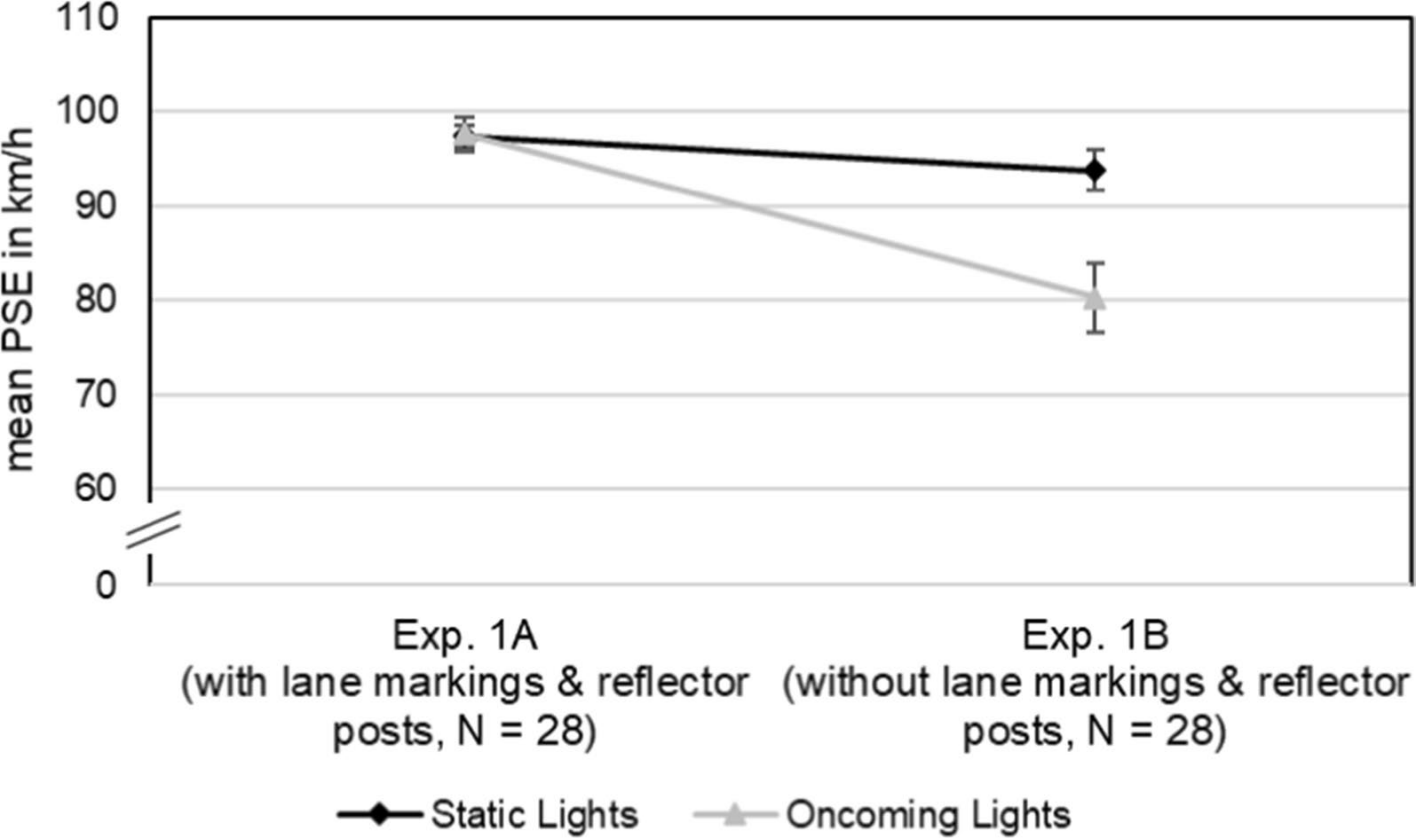
Mean PSEs (in km/h). *Note* Results for static lights and oncoming lights for Experiment 1A (with lane markings and reflector posts) and Experiment 1B (without lane markings and reflector posts). Error bars indicate standard errors

To analyze the interaction effect further, we conducted two separate post hoc t-tests for paired samples (two-tailed), one for Experiment 1A and one for Experiment 1B. The t-test for Experiment 1A revealed no significant difference between static lights (*M* = 97.43 km/h, *SD* = 6.32 km/h) and oncoming lights (*M* = 97.60 km/h, *SD* = 9.36 km/h),* t*(27) =  − 0.15, *p* = 0.879, *d*_*z*_ = 0.03, 95% CI [94.98, 99.88]. The post hoc t-test for Experiment 1B, however, showed that the PSE of static lights (*M* = 93.79 km/h, *SD* = 11.46 km/h) was significantly higher than the PSE of oncoming lights (*M* = 80.32 km/h, *SD* = 19.59 km/h), *t*(27) = 3.05, *p* = 0.005, *d*_*z*_ = 0.58, 95% CI [89.35, 98.24], see Fig. 4.

Results of the post-questionnaire revealed that seventeen participants in Experiment 1A reported to have used the strategy of estimating the traveling speed by counting reflector posts and eight participants said that they had counted the gaps of the road markings in the center of the road (multiple answers possible). Eleven participants in Experiment 1B reported to have used a field on the left side of the road in the simulated environment as a reference to estimate speed and distance driven. Two participants in Experiment 1A and one participant in Experiment 1B mentioned to have counted the seconds of the video sequence. Another strategy used by one participant was to estimate the traveling speed by the distance traveled. One participant in Experiment 1A and one participant in Experiment 1B oriented themselves toward the lights on the sides of the road. One participant in Experiment 1A explained to have tried to find an inner rhythm when watching the video sequence. Four participants in Experiment 1A and fifteen in Experiment 1B reported to not have used any strategy. Every participant confirmed to have seen the lights when asked.

## Discussion

We conducted Experiment 1A and 1B in a laboratory setting to investigate the effect of visuospatial stimuli on speed perception. Our participants encountered a simulated straight road with either static lights or oncoming lights, either with reflector posts and road markings (Experiment 1A) or without road markings (Experiment 1B). We applied the method of constant stimuli (see, e.g., Pretto et al., 2012). Participants had to indicate which traveling speed in two consecutive video sequences they perceived as faster. This led to the calculation of the point of subjective equality (PSE), indicating the speed at which participants perceived the velocity in both video sequences to be the same.

We did not find a difference between static lights and oncoming lights when reflector posts and road markings were present in Experiment 1A, though we expected the oncoming lights to alter the optic flow (Gibson, 1950; Köhler et al., 2022; Manser & Hancock, 2007). It is possible that drivers did not actively notice the moving component of the lights, even though every participant had seen the lights itself.^2^ However, most participants reported to have counted either reflector posts or gaps between road markings. According to Lidestam et al. (2019), this is being taught in some driving lessons to estimate speed and could have influenced our results: All participants reported to have seen the lights and the majority mentioned the movement component. It is therefore possible that those participants who reported to have used this strategy based their responses on their counting strategy alone. It is also possible that actively driving, as participants did in the field test of the study by Köhler et al. (2022), instead of the passive task of watching video sequences could have prevented the use of this strategy due to limited resources. This could be subject to future investigation.

However, we found a significant interaction between presentation condition and presence of road markings, suggesting that perceived speed differed between Experiment 1A and Experiment 1B regarding the two tested presentation conditions, static and oncoming lights. While we did not find a difference between oncoming and static lights when road markings were present in Experiment 1A, post hoc t-tests revealed a significantly lower PSE for oncoming lights than for static lights when road markings and reflector posts were not part of the experimental setting in Experiment 1B. This significant difference between static and oncoming lights in Experiment 1B was 13 km/h, which is considerably more than in Experiment 1A. Due to the significant interaction, we can assume that this difference was significantly bigger in Experiment 1B than in Experiment 1A. Using road markings and reflector posts to estimate speed was likely a well-learned process (Theeuwes, 2021), on which drivers relied in Experiment 1A. When these were not present, drivers relied on other sources of information to estimate their traveling speed, which were the lights on the side of the road.

Interestingly, lights on the side of the road led to a clear difference in perceived speed but had no influence when they were overruled by other processes such as counting reflector posts, as presumably in Experiment 1A. Lidestam et al. (2019) categorized processing of speed-related visuospatial information into energy- and rhythm-based processing. When a vehicle moves at a higher speed in the visual field, it has a higher energy. This is described as energy-based processing. This is like the optic flow described by Gibson (1950). However, continuously placed objects like road markings or reflector posts create a rhythm when driving. The frequency of this rhythm is higher at greater velocities and lower when driving at lower speeds. Lidestam et al. (2019) described rhythm-based processing as more dominant than energy-based processing, and as a result, human speed perception would be likely to rely on the rhythm of distinct objects in the field of view. Therefore, when there are stimuli located closely to the participants, it is more likely that participants perceive speed based on these objects in a rhythm-based manner. This would require some amount of cognitive processing. Consequently, counting reflector posts is likely a process explicitly requiring attention (see Cavanagh, 1992; Lidestam et al., 2019). If participants prioritized the counting of reflector posts or road markings, they allocated more resources to this task. It is possible that the task demands for this were high, resulting in a high workload, which would not have allowed participants to process the visuospatial stimuli in the form of red lights (Stapel et al., 2019). Furthermore, it could be examined further which factors influence drivers speed perception toward a more energy-based processing and which toward a more rhythm-based processing. If lights were, for example, spaced similarly to the reflector posts or the road markings, this could draw upon the same explicit cognitive processing as counting reflector posts and might lead to a greater focus on the light stimuli. To examine whether participants would still apply this technique in more naturalistic environments with more and different stimuli, future studies could determine the influence of the lights when different stimuli allowing for rhythm-based processing are present. Also, future research could gradually add more distractors to the experimental setup to determine the exact influence of such distractors on speed perception. Nevertheless, visual information has been reported to be the primary source of information in driving (Macadam, 2003) and thus limits processing of other input (e.g., Posner et al., 1976). Speed-related information is thus likely processed automatically. While the experimental task of comparing the traveling speed in video sequences could technically have been accomplished without the light, our results suggest that they are still being considered, especially in the absence of additional speed-relevant stimuli. These additional stimuli are such strong predictors of speed that additional information, such as the light stimuli in our study, has little further effect on the perception of traveling speed. However, salient stimuli capture automatic attention even if their information is nominally irrelevant (see, e.g., Lange-Malecki & Treue, 2012). The light stimuli in our study are therefore suitable for quasi-subliminal manipulation in speed perception, which can be more efficient than a mere warning sign (see, e.g., Köhler et al., 2022).

The results provide further insights into potentially underlying perceptual reasons in the countermeasure to speeding developed by Köhler et al. (2022), where the light stimuli of our laboratory study were applied in a field test. While both oncoming and static lights led to lower driving speeds in comparison to a baseline without lights, there was no difference between static and oncoming lights (Köhler et al., 2022). This could have come about due to the warning character of the lights in general (Kahneman, 1973), independent from a movement component. The movement component of oncoming lights might have been overruled by attention to other stimuli in the driving environment. More specifically, problems in information processing especially arise when processes interfere, as human information processing is limited due to crosstalk phenomena (see Koch et al., 2018). The results of the present study suggest that driver’s attention in the field test by Köhler et al. (2022) was occupied with other stimuli. Drivers are therefore unlikely to base their speed estimation on stimuli aiming at influencing the optic flow when other stimuli for this purpose are present.

In the present study, we tested the influence of visuospatial stimuli on human speed perception, because we regarded driving as a spatial control task (see Gibson, 1950; Manser & Hancock, 2007). This paper focused on the input of visual stimuli on speed perception based on an applied measure described by Köhler et al. (2022), who already implemented such a solution in the field. However, according to the theory of directed attention, auditory stimuli are described to attract attention more quickly than visual stimuli (see Aschersleben & Bertelson, 2003; Lukas et al., 2010, 2014). This leaves the question to what extent auditory stimuli play a role in human speed perception as well. In a laboratory test, people who heard more of their car’s engine noise chose to drive slower (Horswill & McKenna, 1999). What remains open, however, is if there might be an acoustic flow (see Pretto & Chatziastros, 2006) when passing objects on the driving environment, similar to the optic flow (Gibson, 1950; Köhler et al., 2022; Manser & Hancock, 2007; Palmisano, 2002).

Note that there are also limitations to our findings and the practical conclusions we wish to draw from them. Firstly, the effect of experiencing slower traveling speeds with oncoming lights in the absence of road markings and reflector posts could have been the result of further learned patterns. Not having lane markings and reflector posts could have been associated with more inhabited surroundings, leading to the expectation of slower movement, as the environment likely influences driving speed (Dumbaugh et al., 2020). Charlton et al. (2010) removed lane markings and adjusted landscaping to mark local roads in a study on self-explaining roads, which led to lower driving speeds. However, this seems unlikely for our study as there were no further cues that could have led to this assumption.

Secondly, speed perception could also be influenced by the field of view, both vertically and in the periphery. According to Lidestam et al. (2019), the bigger the field of view, the slower the chosen driving speed of participants. We placed the participants in our study quite closely in front of a 49″ screen to create some peripheral vision of the experimental stimuli. It is possible that a curved screen would be more suitable to create a higher immersion.

Thirdly, the difference in displayed speed levels to calculate the PSE between Experiment 1A (7 levels) and Experiment 1B (5 levels) could have influenced the results. However, this is unlikely, as the number of levels increases the likelihood to be able to calculate a PSE at all, not where the PSE would be located. Furthermore, one could argue that the speed levels between static lights (70 km/h-130 km/h in Experiment 1A, 80 km/h-120 km/h in Experiment 1B) and oncoming lights (80 km/h-120 km/h in Experiment 1A, 60 km/h-110 km/h in Experiment 1B) might not have been fully comparable. However, the assumption behind this was that the PSE within one level of the factor presentation condition (static lights vs. oncoming lights) is always located in the same spot: The PSE shows where two sequences are perceived as equal. Both static and oncoming lights were compared to the same reference sequence. So even if we had tested speed levels between 70 km/h and 130 km/h for oncoming lights as we had for static lights, we would expect the PSE to be the same because the reference sequence and the test sequence would be perceived as equally fast at the same speed for oncoming lights than when testing speed levels between 50km/h and 110 km/h. However, we wanted to ensure that the traveling speeds we chose would be scattered around the actual PSE. Since we expected the PSE to be lower with oncoming lights, we chose lower driving speeds based on our pretest. This assumption proved to be correct when we found an influence of the oncoming lights on the subjectively perceived traveling speed in Experiment 1B. Here, the PSE for oncoming lights was at 80.32 km/h, which was exactly in the middle of the five provided speed levels. Furthermore, the same speed levels for static and oncoming lights could have led to a virtually perceived imbalance for oncoming stimuli: If our participants had always seen video sequences that they perceived as faster than the reference sequence, they might have felt the need to balance their responses of “faster” or “slower” more. In adjusting the speed levels for test sequences for oncoming lights, we aimed at moderating this potential bias.

Further, placing the lights on the level of the road surface could have been unfamiliar to drivers, as lights are rather placed on delineators or roadside barriers in the driving context than on the ground. It remains open whether placing the lights on the level of delineators would have had a similar effect. However, we would assume the height to be irrelevant, as we saw an effect of the optic flow on the ground level, which is even further away from the focal point. It is therefore likely to find the same effect at a higher position. Another potential limitation could be the different durations of the two studies as Experiment 1B was part of a larger experiment and participants could have experienced fatigue because of spending so much time with the rather repetitive experimental task requiring constant attention. However, we found the significant difference between static lights and oncoming lights in the longer overall experiment. It is therefore unlikely that fatigue affected our results substantially.

Further, while we aimed at comparable samples for Experiment 1A and Experiment 1B and achieved this especially in terms of gender distribution and mean age, we cannot exclude a potential influence of sample differences. Two participants in Experiment 1B did not hold a driver’s license and one person held it for 44 years, leading to a higher standard deviation for the sample of Experiment 1B. After not finding any extreme values in their results, we decided to keep them in the sample, since the experimental task did not include driving itself, but referred to the mere perception of traveling speed, which does not necessarily require active driving experience in a car. However, it is still possible that these sample differences might have had an influence on the results. Future studies could determine the role of driving experience in speed perception, especially since driving experience and expertise in certain tasks have been reported to be qualitatively different when it comes to driving (for an overview, see Pammer & Blink, 2018).

Lastly, our study was conducted under laboratory conditions. While this allows control of potentially confounding variables, it is also not a natural driving situation. Nevertheless, knowledge on the role of different perceptual cues in speed perception can support the development of innovative interventions that aim at enhancing traffic safety and should be investigated further to determine their influence in naturalistic traffic as well.

The findings in this study would allow further application of the tested measure in naturalistic traffic, as done by Köhler et al. (2022). Furthermore, this measure could present an interesting in-car application, for example, by using a head-up display to project such light applications on the road via augmented reality or by animations of ambilight to mimic the optic flow inside the vehicle. However, both applications would require further investigation especially in terms of driver distraction, since applications inside the vehicle must not distract from the driving task or be visually entertaining (European Commission, 2008).

### Conclusion

This paper investigated the effect of visuospatial stimuli in the form of red lights on drivers’ speed perception based on a countermeasure to speeding developed by Köhler et al. (2022) in two experiments. We found that visuospatial stimuli in the form of oncoming lights led to a higher perceived traveling speed than static lights, but only when further visual stimuli such as reflector posts and road markings were removed. Active processes such as counting other stimuli might overrule the automatic processing of the light stimuli. Future research should investigate whether speed perception in the traffic context is a mainly visual task or whether auditory information could have an influence on speed perception as well.

## Data Availability

The datasets used and/or analyzed during the current study are available from the corresponding author on reasonable request.

## Footnotes

1 The long period between Experiment 1A and 1B was due to planning and testing difficulties during COVID-19. For Experiment 1B, we complied with the local COVID-19 regulations in July 2021. Participants were alone in the testing room and were connected to the experimenter via a video conferencing system. We aligned the camera in the test room in such a way that the experimenter could see the test monitor and the participant from behind via the video conferencing system.

2 We investigated whether red lights by themselves might have an influence in a pre-study (not reported) with 27 participants, comparing red static lights with the same specifications as in Experiment 1A to a baseline of video sequences without lights in the driving environment. We did not find a significant difference of PSEs, suggesting that red lights per se did not influence speed perception in our experimental setting.
